# Acquisition of Genetic Aberrations by Activation-Induced Cytidine Deaminase (AID) during Inflammation-Associated Carcinogenesis

**DOI:** 10.3390/cancers3022750

**Published:** 2011-06-22

**Authors:** Atsushi Takai, Hiroyuki Marusawa, Tsutomu Chiba

**Affiliations:** Department of Gastroenterology and Hepatology, Graduate School of Medicine, Kyoto University, 54 Shogoin-Kawahara-cho, Sakyo-ku, Kyoto 606-8507, Japan; E-Mails: atsushit@kuhp.kyoto-u.ac.jp (A.T.); chiba@kuhp.kyoto-u.ac.jp (T.C.)

**Keywords:** activation-induced cytidine deaminase, inflammation, carcinogenesis, genetic alteration

## Abstract

Genetic abnormalities such as nucleotide alterations and chromosomal disorders that accumulate in various tumor-related genes have an important role in cancer development. The precise mechanism of the acquisition of genetic aberrations, however, remains unclear. Activation-induced cytidine deaminase (AID), a nucleotide editing enzyme, is essential for the diversification of antibody production. AID is expressed only in activated B lymphocytes under physiologic conditions and induces somatic hypermutation and class switch recombination in immunoglobulin genes. Inflammation leads to aberrant AID expression in various gastrointestinal organs and increased AID expression contributes to cancer development by inducing genetic alterations in epithelial cells. Studies of how AID induces genetic disorders are expected to elucidate the mechanism of inflammation-associated carcinogenesis.

## 1. Introduction

Chronic inflammation plays a major role in human carcinogenesis [1,2]. The development of hepatocellular carcinoma (HCC) caused by hepatitis B virus or hepatitis C virus (HCV) infection [3,4], gastric cancer caused by *H. pylori* infection [5,6], and colitis-associated cancer caused by ulcerative colitis [7,8] are representative examples of inflammation-associated carcinogenesis. Various inflammatory mediators are highly expressed in inflammatory tissues, and inflammation-triggered anti-apoptosis activity and cell growth cause cancer development [1].

Nuclear factor (NF)-κB is a well-known transcriptional factor that is associated with the pathophysiology of inflammation. NF-κB is activated by various proinflammatory cytokines, such as tumor necrosis factor (TNF)-α, and viral/bacterial infection, leading to the expression of various cytokines and molecules involved in the determination of cell fate [9,10]. Further, NF-κB expression is thought to be deeply involved in the process of inflammation-associated carcinogenesis [11,12]. For example, high expression levels of TNF-α in the liver tissues of patients with chronic viral hepatitis activate the NF-κB classical pathway, which is associated with cell proliferation and suppression of apoptosis, leading to hepatocarcinogenesis [13]. In an animal model of colitis-associated carcinogenesis, NF-κB activation in the chronically inflamed colonic tissues promotes the transcription of apoptosis inhibitory molecules, including BCL-XL and GADD45β [14]. In addition, interleukin (IL)-6 produced by inflammatory cells activates the JAK1-STAT3 pathway via gp130 activation, leading to cell growth [15]. The detailed mechanisms of carcinogenesis in inflammation-associated cancer development, however, remain unknown.

Genetic changes, such as nucleotide alterations and chromosomal translocation occurred in oncogenes and tumor-suppressor genes, have an important role in cancer development [16]. Sequencing of whole genomes, whole exomes, and whole transcriptomes of cancer samples has recently become feasible using second-generation sequencing technologies (also known as next-generation sequencing) [17]. Use of these technologies to analyze the whole genomes of various cancer tissues, such as acute myeloid leukemia, lung cancer, breast cancer, and pancreatic cancer has led to the detection of a variety of nucleotide alterations, gene amplifications, and chromosomal translocations [18-20]. In addition, almost all of the nucleotide alterations are “passenger mutations”, which are not involved in carcinogenesis, in contrast to the small percentage of “driver mutations”, which directly contribute to oncogenesis [21]. On the other hand, organ-specific profiles of copy number variations have been reported in the genome of various cancer tissues, including HCC and lung cancer, based on the traditional comparative genomic hybridization array analysis [22,23]. In some diseases, such as hereditary non-polyposis colorectal cancer, abnormalities in DNA mismatch repair genes lead to the accumulation of nucleotide alterations in various genes and colon carcinogenesis [24,25]. Genetic aberrations in DNA repair systems, however, have been reported in only a few cancers and the molecular mechanism for acquiring the genetic abnormalities remains unclear for most cancers.

## 2. Physiological Roles of Activation-Induced Cytidine Deaminase

Several molecules that possess nucleotide editing activity were recently identified. These molecules are called nucleotide editing enzymes and include the apolipoprotein B mRNA-editing enzyme, catalytic polypeptide-like (APOBEC) family [26]. The APOBEC family molecules are thought to have an important role in maintaining homeostasis and the immunologic response by inducing somatic mutations in targeted DNA or RNA sequences. For example, APOBEC1 contributes to the regulation of lipid metabolism by inducing nucleotide alterations at specific sequences of mRNA transcribed from the *apoB* gene [27-29]. On the other hand, APOBEC3G has antiviral activity against a broad range of retroviruses, including human immunodeficiency virus, for its DNA editing potential in the nascent retroviral of DNA [30-32]. Among the APOBEC family molecules, only activation-induced cytidine deaminase (AID) induces genetic changes in human DNA sequences.

**Figure 1.**
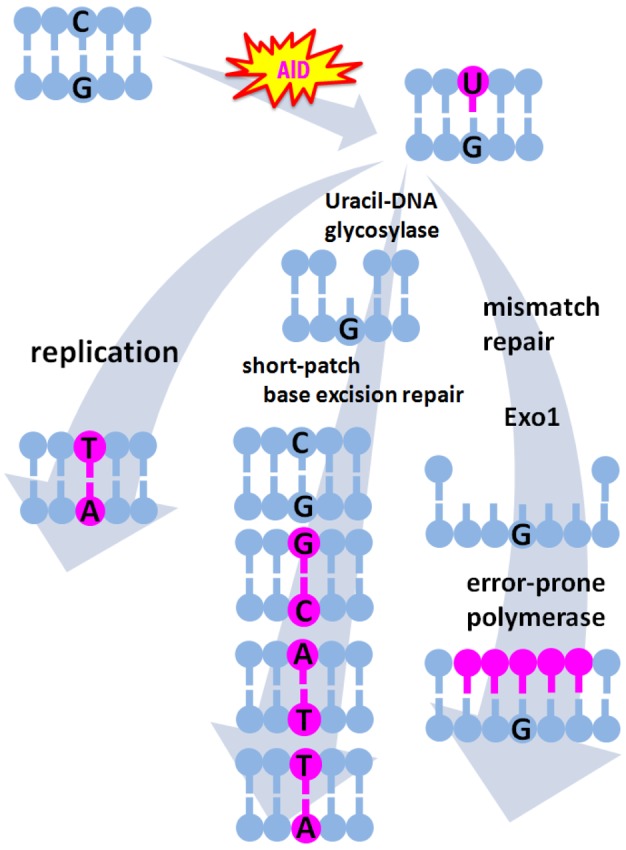
Activation-induced cytidine deaminase (AID) induces genetic changes in human DNA sequences. AID converts cytosine (C) to uracil (U) on the target DNA sequence, creating U-guanine (G) mismatch that is resolved by several pathways. (Left pathway) The general replication machinery interprets U as if it was thymine (T), generating C to T and G to adenine (A) mutations. (Middle pathway) Uracil-DNA glycosylase removes an U nucleotide, creating an abasic site. Short-patch base excision repair fills the gap with error-prone polymerases, which can insert any nucleotide in place of the U nucleotide, leading to both transition and transversion mutations at C-G pairs. (Right pathway) Mismatch repair recognizes U-G mismatch. U-bearing strand is excised by Exo1 and error-prone polymerases fill the gap, leading to mutations at A-T pairs as well as at neighboring C-G pairs.

AID is expressed only in activated B cells under physiologic conditions and contributes to two unique molecular mechanisms for antigen-driven immunoglobulin (Ig) gene diversification. These mechanisms include somatic hypermutation (SHM) and class switch recombination (CSR). SHMs are point mutations introduced into the variable (V) region of Ig gene at a high frequency, leading to the production of a variety of high-affinity antibodies [33,34]. AID converts cytosine (C) to uracil (U) on the sequence of the V region of Ig gene [33,35]. U-guanine (G) mismatch created by AID is resolved by several pathways that may compete with one another (Figure 1). If the resulting U-G mismatch is not repaired before the onset of DNA replication, DNA polymerase will insert an A nucleotide opposite the U nucleotide, generating a C to thymine (T) and G to adenine (A) transition [36]. Alternatively, removal of the U nucleotide by uracil-DNA glycosylase creates an abasic site, which gives rise to both transition and transversion mutations at C-G base pairs when a short-patch base-excision repair can fill the gap with error-prone polymerases [37]. On the other hand, the mismatch repair heterodimer Msh2/Msh6 is thought to trigger the excision and error-prone resynthesis of DNA sequences, leading to mutations at the A-T base pairs near the initiating U-G mismatch. Indeed, more than half of the mutations accumulated in the Ig V region are in A-T bases, which are not the result of the direct biochemical action of AID [38,39]. In contrast to SHM, CSR is a unique type of intrachromosomal deletional recombination that replaces the constant (C) region of the Ig heavy chain, allowing the expression of antibodies that have the same antigen specificity but are a secondary IgH isotype (*i.e.*, IgG, IgA, or IgE) and thereby have different effector functions. The cytidine deaminase activity of AID has an essential role in SHM as well as in the production of DNA double strands breaks that occur in CSR [33,35,40].

The precise mechanisms of how AID selects the target genes and target sequences still remain unknown at present. However, it has been shown that AID preferentially deaminates C nucleotides in so-called WRCY motifs (where W = A or T, R = A or G, and Y = C or T) in transcribed targets [41-43]. Pham *et al.* examined these mutational hotspots and revealed AID targeting of these sequences; 14 of 15 targets were recognized as WRCY motif sequences [44]. When we consider the characteristics of SHM in the V region, most nucleotide alterations are single-base changes that occur starting 100 to 200 bp from the transcription initiation site and end 1.5 to 2.0 kb downstream [37,45-48]. In addition, mutation frequency is proportional to the rate of transcription [49-51].

## 3. Roles of Activation-Induced Cytidine Deaminase in Inflammation-Induced Carcinogenesis

### 3.1. Genetic Alteration Induced by AID Causes Tumorigenesis

The activity of AID as a genome mutator raises the question of whether AID induces inappropriate mutations in non-Ig genes. Recently, Liu *et al.* performed extensive sequencing of Ig and transcribed non-Ig genes in germinal center B cells from mouse Peyer's patches [52,53]. Approximately 25% of the expressed genes analyzed accumulated statistically significant levels of nucleotide alterations in an AID-dependent manner [53]. These findings indicate that AID acts broadly on the genome, but mutations induced by AID occur at different rates in each gene. Sequence analysis in 83 transcribed non-Ig genes extracted from Ung/Msh2 double-knockout germinal center B cells revealed that more than half of the genes exhibited a strong bias for C:G to T:A transition mutations and enrichment for mutations in AID hotspots [53]. Interestingly, some genes, such as *Myc* and *H2afx*, frequently hit by AID in Ung/Msh2 double knockout B cells are not necessarily mutated at high rates in wild-type B cells. These results indicate that a number of non-Ig genes are targeted by AID but some are protected from substantial mutation accumulation by high-fidelity repair through the combined action of Ung and Msh2, although some other genes are repaired in an error prone manner, frequently leading to accumulation of mutations like Ig gene.

The impact of AID expression in mutagenesis of non-*Ig* genes was clarified by the analyses of mouse models with constitutive AID expression. Okazaki *et al.* demonstrated that all mouse lines with constitutive and ubiquitous AID expression invariably developed T cell lymphomas [54]. They also reported that point mutations are massively introduced in various non-*Ig* genes, including the proto-oncogene *c-myc* and T cell receptor gene, in lymphoma cells. Interestingly, these AID transgenic (Tg) mice develop not only malignant lymphomas but also various epithelial tumors such as liver cancer, lung cancer and gastric cancer [54-56]. Notably, organ-specific preferences for nucleotide alterations are observed in some of the tumor-related genes in each epithelial tissue of the AID Tg mice [55]. For example, *Myc* and *Kras* genes are frequently mutated in lung and stomach tissues of the AID Tg mice, respectively. In contrast, nucleotide alterations in the *Trp53* and *Ctnnb1* genes are commonly induced during the development of lung, liver, and gastric cancers [55]. These findings suggest that inappropriate or deregulated AID expression increases the mutation rate of genes that are not normally attacked by AID and contribute to tumor development in both lymphoid and non-lymphoid organs, and that AID might be involved in the generation of organ-specific genetic diversity in oncogenic pathways during cancer development.

### 3.2. AID and Hematopoetic Malignancy

A number of studies demonstrated high AID expression in various neoplasms of B lymphocytic lineage and determined that AID expression levels are associated with unfavorable gene mutations and chromosomal translocations [57-59]. As described above, it has been reported that mouse models with constitutive expression of AID invariably develop T cell lymphomas [54], and development of B cell lymphoma is observed following transplantation of bone marrow cells from AID Tg mouse **[**60]. Interestingly, AID deficiency reduces the risk for development of Bcl6-dependent germinal center-derived lymphoma, while the loss of AID has no impact on Myc-driven, pre-germinal center lymphomas [61] or on the progression of germinal center-like lymphomas in Msh6-deficient mice [62]. On the other hand, it is well established that AID is required for *IgH-myc* translocation during development of Burkitt's lymphoma and diffuse large B cell lymphoma [63,64]. Ramiro *et al.* provided direct evidence that AID promotes chromosomal translocation between *c-myc* and the *Ig* switch region DNA in normal B cells [65]. In addition, AID protein can be detected in germinal center centroblasts and their transformed counterpart, Burkitt's lymphoma, but not in pre-GC neoplasms, including B cell chronic lymphocytic leukemia [59]. These data suggest that AID is involved in the development and progression of human B lymphocytic neoplasms.

### 3.3. AID and Gastrointestinal Cancer

A causal relationship between inflammation and cancer development is proposed in a variety of chronic inflammatory diseases. In particular, many cancers of gastrointestinal organs, some of which are caused by infectious agents, are known to arise in a background of chronic inflammation. On the other hand, the major transcription factors that mediate AID expression in B cells include NF-κB, STAT6, and the Smad proteins [66]. The signaling pathway that stimulates the activation of these transcription factors is deeply involved in a variety of inflammatory responses associated with carcinogenesis in epithelial organs. These findings support the hypothesis that inflammatory stimulation of epithelial cells could induce the aberrant AID expression and initiate and/or promote oncogenic pathways by enhancing susceptibility to mutagenesis.

#### 3.3.1. Hepatocellular Carcinoma

Epidemiologic studies demonstrate that most human HCC develops during chronic hepatic inflammation associated with liver cirrhosis or chronic hepatitis [3]. On the other hand, nucleotide alterations frequently occur in tumor-related genes, such as *TP53* in HCC tissues [67]. In addition, various genetic alterations accumulate in various genes in the chronic hepatitis tissues before HCC develops [68]. These findings indicate that genetic alterations gradually accumulate in chronically inflamed liver tissue.

**Figure 2.**
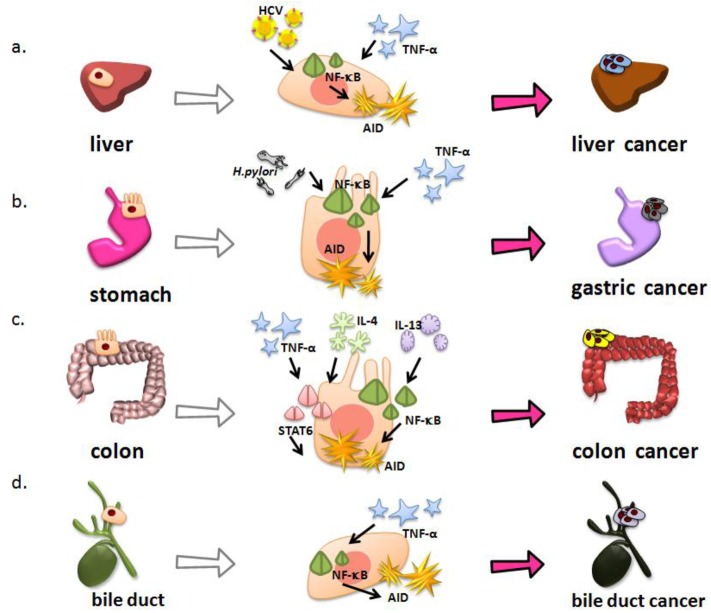
AID expression induced by chronic inflammation causes the development of cancer in various gastrointestinal tissues via its mutagenic activity. **(a)** In hepatitis C virus (HCV)-infected liver tissues, both chronic inflammatory stimulation and direct effects of the HCV core protein cause aberrant AID expression via NF-κB activation, leading to the development of hepatocellular carcinoma (HCC). **(b)** AID is ectopically expressed in *Helicobacter pylori*-infected gastric epithelial cells via nuclear factor κB (NF-κB) activation and causes the development of gastric cancer. **(c)** In the patients with inflammatory bowel disease, AID is upregulated in chronically inflamed colonic epithelial cells not only via the NF-κB pathway, but also via the STAT6 activation pathway and its genotoxicity causes colitis-associated cancer (CAC) development. **(d)** Aberrant AID expression is induced by chronic cholangitis via NF-κB activation pathway, leading to cholangiocarcinogenesis.

AID is aberrantly expressed in the hepatocytes of chronic hepatitis and liver cirrhosis tissues caused by HCV infection, although AID should be expressed only in activated B lymphocytes under physiologic conditions [68]. Endo *et al.* demonstrated that TNF-α stimulation induces AID expression in primary cultured human hepatocytes [69]. AID upregulation in inflamed hepatocytes is achieved through NF-κB activation, because AID protein expression is almost completely abolished by co-production of the super-repressor form of IκBα, a specific NF-κB inhibitor [69]. On the other hand, HCV genome encodes a polypeptide precursor consisting of about 3,010 amino acid residues, and this precursor protein is cleaved by the host and viral proteases to generate at least 10 functional protein units: the core, envelope 1 (E1), E2, p7, nonstructural protein 2 (NS2), NS3, NS4A, NS4B, NS5A, and NS5B [70-74]. The previous studies demonstrated that the core among HCV proteins suppresses apoptotic cell death via the mechanism dependent on the activation of NF-κB pathway [75]. Consistent with these previous findings, AID expression is observed in cultured human hepatocytes expressing HCV core protein via activation of NF-κB pathway [69]. These findings suggest that AID is aberrantly upregulated in human hepatocytes, both by chronic inflammation and directly by HCV infection, leading to the development of HCC (Figure 2a).

Recent stem cell biology studies suggest that the ability to maintain tumor formation/growth specifically resides in a small population of cells called cancer stem cells. The cancer stem cell theory suggests that a tumor comprises a heterogeneous population of cells that form a distinct cellular hierarchy; only a subset of cells within this tumor hierarchy has the ability to initiate and sustain tumor growth. Each of the small subset of cancer stem cells in the tumor has a significantly higher probability of becoming a tumor-founding cell relative to the non-cancer stem cells that make up the bulk of the tumor [76]. According to this theory, it is thought to be effective to identify the cancer stem cells responsible for tumor initiation and progression as therapeutic targets. Many studies have demonstrated that cancer stem cells exhibit many classical properties of both normal stem cells and cancer stem cells, including the following: (i) a high self-renewal capacity; (ii) an ability to differentiate to heterogeneous lineages; (iii) an increased capacity for self-protection against drugs, toxins and radiation; (iv) a capacity to initiate and sustain tumor growth [77]. However, despite their resemblance to the normal stem cells, the exact origin of cancer stem cells is still unclear [78]. The question of whether cancer stem cells are transformed from normal stem cells, or whether they originate from more differentiated cells at a lower level of cellular hierarchy remain to be elucidated for a better understanding of the origin and role of this special subpopulation of cells.

Tissue non-specific alkaline phosphatase (TNAP) is a marker of embryonic stem cells and immature stem cells, and is expressed in murine fetal liver tissues [56]. TNAP-AID mice, in which AID is expressed in cells that produce TNAP, develop HCC at a high frequency [56]. Furthermore, a high incidence of mutation accumulation in the *Trp53* gene is observed in both HCC tissues and non-cancerous liver tissues in TNAP-AID mice [56]. These results indicate that the AID expression in immature hepatocytes contributes to the development of HCC via the accumulation of genetic alterations.

#### 3.3.2. Gastric Cancer

*Helicobacter pylori (H. pylori)* infection causes chronic gastric inflammation and is defined as a class one carcinogen for human gastric cancer [5,79]. Matsumoto *et al.* detected that the ectopic AID production in both gastric cancer tissues and chronic gastritis tissues infected with *H. pylori*, although no AID expression was observed in normal gastric mucosa or in gastric tissues after eradicating the *H. pylori* [80]. These findings suggest that *H. pylori* infection induces aberrant AID expression in gastric epithelial cells. *H. pylori* can be subclassified into “*cag*” pathogenicity island (*cag*PAI)-positive and *cag*PAI-negative strains based on the presence or absence of *cag*PAI, a 40-kb genome fragment containing 31 genes [81]. The *cag*PAI-positive isolates are more virulent strains that produce severe pathologic infection in humans [82], and several studies have provided evidence linking *cag*PAI- positive strains to an increased risk of gastric cancer [83,84]. Interestingly, AID expression is induced by *cag*PAI-positive *H. pylori* infection in gastric epithelial cells, while *cag*PAI-negative *H. pylori* infections have little effect on endogenous AID expression [80]. On the other hand, *cag*PAI-positive *H. pylori* infection is associated with increased activation of NF-κB in gastric epithelial cells both *in vitro* and *in vivo* [85,86]. Intriguingly, *cag*PAI-positive *H. pylori* infection induces aberrant AID expression in gastric epithelial cells via NF-κB activation. Together, these findings suggest that *cag*PAI-positive *H. pylori* directly induces AID expression via NF-κB activation, and that the proinflammatory response caused by *H. pylori* infection also triggers AID expression via the activation of NF-κB in gastric epithelium, because proinflammatory cytokines, such as TNF-α, can induce NF-κB activation in various types of cells. In *in vitro*-cultured gastric epithelial cells, *cag*PAI-positive *H. pylori* infection led to somatic mutations in the tumor-suppressor *TP53* gene. The number of nucleotide alterations observed in *H. pylori-*inf*ected* cells was significantly reduced by knockdown of endogenous AID, indicating that the somatic mutations in the *TP53* gene in *cag*PAI-positive *H. pylori* infected cells were due to the induction of endogenous AID expression in gastric cells. These findings indicate that *cag*PAI-positive *H. pylori* infection causes accumulation of somatic mutations in tumor-related genes such as *TP53* through aberrant AID upregulation in gastric epithelial cells. Recent study further revealed that reduction of copy numbers of specific gene loci such as *CDKN2A, CDKN2B,* and *BCL6* occurs in gastric epithelial cells with aberrant AID expression [80,87]. In contrast, these genetic abnormalities are not observed in AID-deficient gastric epithelial cells [80,87]. These findings provide evidence that the ectopic AID expression caused by *H. pylori* infection might be a mechanism for producing genetic aberrations in the gastric mucosa during *H. pylori*-associated gastric carcinogenesis (Figure 2b).

#### 3.3.3. Colitis-Associated Colorectal Cancer

The incidence of colorectal cancer is significantly higher in patients with inflammatory bowel disease including ulcerative colitis (UC) than in the general population [7]. This type of colorectal cancer is called colitis-associated colorectal cancer (CAC) and is different from sporadic colorectal cancer that originates from colorectal adenomatous polyps in the molecular pathogenesis of cancer development. In sporadic colorectal cancer, colorectal adenoma first develops through the occurrence of an *APC* gene mutation, and other genetic alterations such as *KRAS* activation and *TP53* inactivation occur during carcinogenesis [88,89]. In contrast, mutations in the *TP53* gene appear to be an early event and already present in the colonic mucosa of patients with UC before CAC onset [90-92]. Interestingly, aberrant endogenous AID protein is detected both in the inflamed colonic mucosa and CAC tissues of UC patients [93]. In addition, AID expression is induced by TNF-α stimulation in colonic cells via the NF-κB pathway, which is constitutively activated in the colonic epithelia of patients with inflammatory bowel disease [93,94]. On the other hand, colonic mucosal inflammation is usually mediated by either an excessive T helper cell (Th) 1 T-cell response associated with increased interferon-γ and IL-12, or an excessive Th2 T-cell response associated with increased IL-4 and IL-13 secretion [95,96]. Although the concentration of the Th2 cell-driven cytokine IL-4 varies in UC colon tissue, UC is considered to have a Th2 profile [95,97]. Indeed, a recent study in mice suggests that production of IL-13 is an important pathologic factor for UC [98]. Moreover, UC has an atypical Th2 response, mediated by natural killer T-cells that secrete IL-13 [99], and markedly elevated levels of IL-13 production are observed in UC patients [100]. These Th2 cytokines regulate various molecules via the transcriptional factor STAT6 activation [100-102]. Both IL-4 and IL-13 can induce AID expression via the STAT6 pathway in colonic cells [93]. These findings indicate that AID expression in colonic epithelial cells is regulated not only via the NF-κB pathway but also via the STAT6 activation pathway. Furthermore, a number of genetic alterations are detected in the *TP53* gene in colonic cells that have continuous AID expression, whereas no significant mutations are observed in *APC* and *KRAS* genes [93]. These results suggest that the proinflammatory cytokine-mediated aberrant AID expression in colonic epithelial cells is a genotoxic factor linking inflammation, *TP53* mutation and CAC development (Figure 2c).

#### 3.3.4. Cholangiocarcinoma

The incidence of cholangiocarcinoma is the second-most common primary hepatobiliary malignancy [103]. Although most cholangiocarcinoma arise in the absence of apparent risk factors, chronic inflammation of the biliary epithelium has a critical role for their development [104]. In fact, primary sclerosing cholangitis (PSC) is the most common predisposing condition for cholangiocarcinogenesis, and the prevalence of cholangiocarcinoma in patients with PSC is high, ranging from 9% to 23% [103]. Other risk factors for cholangiocarcinogenesis are also associated with chronic biliary tract inflammation, including chronic choledocholithiasis, liver fluke infestation, hepatolithiasis, and HCV infection [104]. Similar to other inflammation-associated gastrointestinal cancers, AID is aberrantly expressed in the tissues of chronic cholangitis and cholangiocarcinoma [105]. In addition, ectopic AID production is induced in response to TNF-α stimulation via the NF-κB activation pathway in human cholangiocarcinoma-derived cells [105]. Furthermore, the aberrant AID expression in biliary cells results in the generation of mutations in tumor-related genes including *TP53* and *INK4A/p16*, both of which are frequently mutated in human cholangiocarcinoma tissues underlying PSC [105-107]. These findings suggest that AID production aberrantly induced by chronic bile duct inflammation causes cholangiocarcinogenesis via the accumulation of genetic aberrations (Figure 2d).

## 4. Conclusions

Until recently, the precise molecular mechanism underlying cancer development due to the chronic inflammation has been unclear. Recent studies indicate the possibility that the mutagenic activity of AID causes inflammation-associated carcinogenesis in various tissues. However, the mechanisms of how the selection of the target sequences is achieved by AID in each organ are still unclear. It is hoped that further elucidation of the precise mechanism of the AID-induced accumulation of genetic aberrations will lead to the development of novel strategies for treating cancer.

## Abbreviations

AID: activation-induced cytidine deaminase
APOBEC: apolipoprotein B mRNA-editing enzyme, catalytic polypeptide-like
CAC: colitis-associated cancer
CSR: class switch recombination
HCC: hepatocellular carcinoma
HCV: hepatitis C virus
*H. pylori*: *Helicobacter pylori*
Ig: immunoglobulin
IL: interleukin
NF-κB: nuclear factor κB
PSC: primary sclerosing cholangitis
SHM: somatic hypermutation
TNAP: tissue non-specific alkaline phosphatase
TNF: tumor necrosis factor
UC: ulcerative colitis
